# Efficacy of bakuchiol-garlic combination against virulent genes of C*andida albicans*

**DOI:** 10.7717/peerj.12251

**Published:** 2022-01-03

**Authors:** Ayesha Fahim, Wan Harun Himratul-Aznita, Puteri Shafinaz Abdul-Rahman, Mohammad K. Alam

**Affiliations:** 1 Department of Oral & Craniofacial Sciences, Faculty of Dentistry, University of Malaya, Kuala Lumpur, Malaysia; 2 Department of Oral Biology, University College of Dentistry, University of Lahore, Lahore, Pakistan; 3 Department of Molecular Medicine, Faculty of Medicine, University of Malaya, Kuala Lumpur, Malaysia; 4 University of Malaya Centre for Proteomics Research, University of Malaya, Kuala Lumpur, Malaysia; 5 Orthodontic Division, Preventive Dentistry Department, College of Dentistry, Jouf University, Jouf, Saudi Arabia

**Keywords:** Bacteria, Fungus, Alternative medicine, Natural product, Dentistry, Oral biology

## Abstract

**Background:**

Polymicrobial biofilms are notorious for causing intraoral tissue destruction. *Streptococcus sanguinis* and *Streptococcus mitis*, commensals of oral cavities, have been found co-existing with *C. albicans* in resistant oral infections. There is an urgent need to find alternative treatment options. This study aims to assess the efficacy of garlic (G) and *bakuchiol* (Bk) combination against candida virulent genes and their subsequently secreted proteins.

**Methods:**

*In vitro* single species biofilms of *C. albicans*, and mixed species biofilms formed in combination with streptococci were exposed to *bakuchiol* and garlic extract (Bk+G). Gene expression of agglutinin-like sequence (*ALS1), (ALS3)*, adhesin-like wall proteins *(HWP1)* and aspartyl proteinases (*SAP5)* were determined using qPCR and their subsequent proteins were assessed through Western blotting.

**Results:**

Virulent genes were significantly downregulated in single species biofilms when they were treated with Bk+G combination. However, Bk+G did not have significant effect on *ALS1* and *HWP1* gene in polymicrobial biofilms. *ALS3* and *SAP5* were significantly downregulated in Bk+G treated polymicrobial biofilm. Similar results were portrayed in Western blotting.

**Conclusion:**

Bk+G combination exhibited antimicrobial effects against single and mixed species biofilms. The findings might provide insights for treating resistant candida infections. This combination could potentially serve as an herbal alternative to traditional antifungals following further research.

## Introduction

*Candida albicans* is an opportunistic fungus of the oral cavity that causes challenging infections alone and in combination with other microorganisms (Swidergall et al., 2019). *Streptococcus sanguinis* and *Streptococcus mitis* although occur in oral cavity as commensals, have been found co-existing with *C. albicans* in various diseases and on several biomaterials like dentures, implants, feeding tubes, voice prostheses and catheters (Montelongo-Jauregui et al., 2019). Candida species not only adheres to oral tissues but requires firm attachment with other microbes as well (Nikou et al., 2019). In order to achieve this, *C. albicans* cell wall utilizes adhesins, amongst which, agglutinin like sequence *ALS*, adhesin-like hyphal wall proteins *HWP* and aspartyl proteinases *SAP* genes have proven to be most virulent during polymicrobial infections (Cavalcanti et al., 2016). *ALS*1 not only mediates attachment of *C. albicans* to host tissue, it undergoes a heterotypic interaction with *ALS*3 between the surfaces of adjacent cells to maintain the integrity of a developing biofilm (Ardehali et al., 2019). *ALS3* plays an important role in biofilm formation, adhesion to host tissue, iron acquisition and invasion of host cells (Deo & Deshmukh, 2019). *SAP5* is famously known to be a proteinase that mediates fungal adhesion and colonization of human oral tissues (Juvêncio da Silva et al., 2021). Expression of *HWP1* has been linked directly with increased biofilm formation and epithelial invasion of *C. albicans* (Goulart et al., 2018). The expression of these genes is known to be enhanced in *C. albicans* polymicrobial biofilms (Ardehali et al., 2019). The growth exhibited by polymicrobial species not only alters the implicit characteristics of the organisms, but it results in failure of traditional therapeutics (Chinnici et al., 2019; Arzmi, Dashper & McCullough, 2019). When polymicrobial infections are not cured by conventional antifungals, doctors switch to harsher options like increasing dosage and/or combining azoles with prednisolone, which not only treats the disease but produces long lasting harm to the human body (Costa-Orlandi et al., 2017). The urgent pharmacological need includes the development of alternative drugs that are more efficient and tolerant than those traditionally in use.

Natural products from plants, animals and minerals have been used as medicines because of their antifungal and antibacterial properties and lesser side effects on human health. *Bakuchiol* (Bk), derived from leaves of *Psoralea glandulosa* (Culen), is being used in India and China for treating skin conditions caused by fungi and bacteria. Garlic (G) (*Allium Sativum*), a part of the *Liliaceae* family, displays antibacterial properties against common pathogenic microorganism (Ibrahim et al., 2020; Jamshidi-Kia, Lorigooini & Amini-Khoei, 2018). To increase the pharmaceutic efficacy of a reagent, and to decrease side effects, it is a common practice in modern medicine to prepare medicaments in combination. In our previous study, it was observed that Bk+G proved to be effective against candida-bacteria biofilm growth with MIC value of 8 + 12.5 *µg/mL* (Fahim, Himratul-Aznita & Abdul-Rahman, 2020). The aim of the current study is to assess the efficacy of the Bk+G combination against the properties that the proteins encoded by the four genes; *ALS1, ALS3, HWP1* and *SAP5* give to *C. albicans*.

## Materials and methods

### Strains and chemicals

The study was conducted in Balai Ungku Aziz Lab from August 2020 until January 2021. The fungal strain used was *C. albicans* (ATCC 14053), and bacterial strains *S. mitis* (ATCC 49456) and *S. sanguinis* (BAA 1455) were used to form *in vitro* biofilms. Tryptic Soy Broth (Merck) was used as nutrient media. Artificial saliva (Fisher Scientific) was used for the formation of biofilms.

Bakuchiol comes from *Psoralea corylifolia* L. seed extract (Nordin, Abdul Razak & Himratul-Aznita, 2015). For this *in vitro* study, bakuchiol (purity: ≥95% HPLC) (ChromaDex Inc., Los Angeles, CA, USA) was dissolved in 1% v/v dimethyl sulfoxide (DMSO) (a stock concentration of 1,000 *μg/m*L) and stored at −20 °C until use. Garlic bulb oil (purity: ≥95% HPLC) was purchased from Millipore-Sigma Inc.

### Formation of biofilm

*In vitro* biofilms were formed on glass beads which mimic tooth surface, following the Nordini’s Artificial Mouth model (NAM) protocol (Rahim et al., 2008). A steady salivary flow and constant temperature of 37 °C was provided to form salivary pellicle. Microorganisms were allowed to grow on the salivary pellicle for 24 h to form biofilm. The experimental group (single species and mixed species) was subjected to the MIC concentration of Bk+G combination; 8 + 12.5 µg/mL respectively throughout the experiment, whereas control group (single and mixed species) was not subjected to any treatment.

### Genomic analysis

#### RNA extraction

Expression of *ALS1*, *ALS3, SAP5* and *HWP1* genes was analyzed in 24 h salivary biofilms of single (*C. albicans* alone) and mixed species (*C. albicans* with *S. mitis* and *S. sanguinis*) treated with Bk+G. Oral biofilms were developed on glass cover slips which were placed in the 6-well microtiter plates. A 100 µL inoculum of each, single species and mixed species at ratio of 1:1:1 (concentrations of 1 × 10^6^ cells/mL) was prepared in Tryptic Soy Broth (TSB) and poured respectively in 6-well plates, followed by 100 µL of Bk+G (8 + 12.5 µg/mL). Nutrient broth, without Bk+G was used as positive control. After 24 h incubation, biofilm cells were collected and transferred to 1.5 mL tubes. For RNA extraction, easy-REDTM total RNA extraction kit was used. After addition of pre-lysis buffer, tube was incubated (95 °C for 3 min). Easy-REDTM solution and chloroform were added consecutively, vortexed and incubated (room temperature for 5 min). The aqueous phase was pipetted out after centrifugation, treated with 1 mL cold isopropanol (20 °C for 15 min), washed with ethanol (70%). To avoid DNA contamination, 1–5 µg of total RNA was added to 1 µL of DNAse I buffer and DEPC treated water to get 10 µL solution. The mixture was incubated for 15 min, 1 µL of RNAse-free 20 nM EDTA was added and the mixture heated to 65 °C for 10 min. RNA was dissolved in RNAse free water and checked with spectrophotometer Nanodrop 2,000 (Thermo Scientific) to determine the purity of extracted RNA (A260/280 ratio ~1.8–2).

#### Synthesis of complementary DNA (cDNA)

cDNA was synthesized in Thermocycler (Eppendorf Mastercycler gradient) using 1-step RT-PCR kit (SuprimeScript RT-PCR kit and Premix 2X). Reverse transcription reactions were carried out by adding total RNA template, random primers (1 µL of 50 *µg/m*L) and ingredients from RT-PCR kit in RNAse free water (total volume of 25 µL). The final reaction mix was then incubated (70 °C for 5 min, 37 °C for 60 min). The resultant cDNA was stored in freezer at −20 °C.

#### Quantification of genes using qPCR

Primers used for analysis were designed using Primer3 software (Table 1). Quantitative PCR was performed in a Fast Realtime PCR instrument (Applied Biosystems 7,500) using 96-well qPCR plates as triplicates. Each 20 µL reaction mix consisted of Titan HotTaqEvaGreenVR qPCR mix SYBR-Green PCR Master Mix (10 µL), primer (1 µL of 10 *mmol/L*), cDNA (2 µL) and RNAse free water (6 µL). Thermal cycler protocol consisted of initial denaturation step (95 °C for 2 min), followed by 40 cycles of denaturation (95 °C for 15 s), then primer annealing (58 °C for 30 s) and primer extension (72 °C for 30 s). A final extension (72 °C for 2 min) was conducted followed by freezing (4 °C). A melting curve was generated after the dissociation step (60 °C). Relative gene expression analysis was achieved according to the double delta Ct (threshold cycle) analysis (DDCt) method (Bustin et al., 2009). The expression was normalized to the standard *ACT1* gene.

**Table 1:** Forward (F) and reverse (R) primers used for qPCR analysis.

Gene name	Sequence (5′ $\longrightarrow$3′)	Product size (bp)	Accession no.
ALS1	**F**-GAC TAG TGA ACC AAC AAA TAC CAG A **R**-CCA GAA GAA ACA GCA GGT GA	318	L25902
ALS3	**F**-CCACTTCACAATCCCCATC **R**-CAGCAGTAGTAGTAACAGTAGTAGTTTCAT C	342	U87956
SAP5	**F**-CCTTCTCTAAAATTATGGATTGGAAC **R-**TTGATTTCACCTTGGGGACCAGTAACATTT	231	Q5ABW5
HWP1	**F**-CCATGTGATGATTACCCACA **R**-GCTGGAACAGAAGATTCAGG	572	EU044787
ACT1 *C. albicans* actin housekeeping gene	**F**-CCAGCTTTCTACGTTTCC **R**-CTGTAACCACGTTCAGAC	200	HM997110

### Protein analysis

#### Total protein extraction

For protein extraction, ReadyPrep™ Protein extraction kit (BIO-RAD, Hercules, CA, USA) was used. Microbial cells were scrapped off from glass coverslip biofilms and transferred to 50 mL falcon tubes containing TSB. Suspension was centrifuged at approx. 3,000×*g* for 5 min at 4 °C and pellet was re-suspended in lysis buffer reagent. With the sample on ice, the suspension was sonicated with an ultrasonic probe to disrupt the cells and fragment the genomic DNA. The tube was then centrifuged again, and the pellet diluted in a beaker containing 60 mL of the ice-cold membrane protein concentrating reagent. The suspension was slowly stirred on ice for 60 min. The sample was then centrifuged at 100,000×*g* for 60 min at 4 °C to pellet the membranes and membrane proteins. Each pellet was washed briefly with 3 mL of cold lysis buffer and incubated on ice for 3 min. The membrane-protein pellet(s) from each extraction were re-suspended in 1.0–2.0 mL of complete 2-D rehydration/sample buffer and vortexed to solubilize protein in the buffer. Sample was centrifuged at 16,000×*g* for 15 min at 20 °C. Supernatant was removed and ready for determination of protein concentration.

#### Total protein quantification

Total extracted protein from each sample was quantified using Bio-Rad protein assay kit. Bovine serum albumin (BSA) protein was used as standard. Five dilutions of the extracted sample proteins were prepared. Then, 10 µL of each protein standard and sample was pipetted in 96-well microtiter plate wells. Following which, 200 µL of diluted dye reagent was dispensed in each well and mixed. Plate was incubated at RT for 15 min. Absorbance was measured using a microtiter plate reader (595 nm) (FC-Bios µQuant). Protein lysate was quantified in µg/mL of solution. Protein solutions were assayed in triplicate. For long term storage, protein samples were aliquoted and stored at −20 °C.

#### Protein separation by gel electrophoresis

To reduce and denature samples, separation by their molecular weight was carried out (Márkus et al., 2017). Each lysate was diluted in equal volume of 2X Laemmli loading buffer. Each sample was then boiled for 5 min at 95 °C to denature proteins and centrifuged at 16,000×*g* for 1 min. SDS-PAGE (discontinuous) was performed in a commercially available polyacrylamide mini-gel (8.6 × 6.7 cm) format on 12.5% w/v separating and 4% w/v stacking slab gel at a constant voltage of 65 V for 1 h. Three or more gels were prepared for simultaneous electrophoresis. Each lane contained 20 μg of protein of an extract preparation along with pre stained molecular weight ladder (Precision Plus Protein™ Standards) to determine the expected molecular weight of proteins.

#### Protein transfer from gel to membrane

The gel was removed from cassette and placed in 1× Western transfer buffer (composition: For 2 liters: 125 mM Tris (6.06 g), 192 mM glycine (28.8 g), 20% methanol (400 ml) and deionized water) for 15 min. Filter paper and nitrocellulose (NC) membrane (Sigma-Aldrich, St. Louis, MO, USA) were activated by soaking in transfer buffer for 10 min. Transfer sandwich was prepared by stacking sponge, filter paper, nitrocellulose membrane, gel, filter paper and sponge in a cassette. The cassette was placed in tank and cooled using ice pack. The transfer was performed at constant voltage of 100 V for 30 min.

#### Immunoblotting

The proteins were transferred to nitrocellulose membrane. The NC membrane was rinsed thrice in Phosphate-buffer saline (PBS) to remove excess gel pieces. All areas of membrane that do not contain protein were blocked by 5% BSA in Tris-Buffered Tween Saline (TBST) solution at room temperature for 1 h to prevent nonspecific binding of antibody and to reduce overall background signal.

Primary antibodies *i.e*., anti-als1, anti-als3, anti-sap5 and anti-hwp1 (source*: E.coli*) (Creative bio-labs, Shirley, NY, USA) were diluted in blocking buffer at 1:1,000. NC was placed in diluted primary antibody and incubated overnight at 4 °C. The blot was then rinsed 5 times with TBST for 5 min each to remove stringent background signals. Secondary antibodies: goat anti-rabbit Horse Radish Peroxidase (HRP) conjugate (ThermoFisher scientific, Waltham, MA, USA) were diluted in blocking buffer at 1:300. The NC membrane was incubated in this solution for 1 h at room temperature. The blot was then rinsed five times with TBST for 5 min each.

#### Imaging and data analysis

Immubolin® Clasico (Sigma-Aldrich, St. Louis, MO, USA), a chemiluminescent substrate was applied to the blot and incubated for 5 min without agitation. Enhanced chemiluminescent (ECL) was decanted and excess solution was removed. The membrane was placed on clear plastic wrap to prevent complete drying. Image was captured and analyzed using software (Gel Doc™ Gel imaging system, Bio-Rad, Hercules, CA, USA). Image J software was used to quantify protein bands on the gel.

### Statistical analysis

All results were computed and expressed as mean ± standard deviation (SD) from three determinations performed in triplicate (*n* = 9). Statistical analyses were performed using IBM SPSS statistics software (version 22.0). The student’s t-test was used to compare readings of genes between control (biofilm without treatment) and experimental (biofilms treated with Bk+G). The one-way ANOVA test was used for comparison among multiple genes within the same group. The least significant difference (LSD) between the means was estimated at the 95% confidence level with the degree of freedom *n*−2. A *p* value of less than 0.05 was considered statistically significant.

## Results

### Genomic analysis

**Figure 1:**
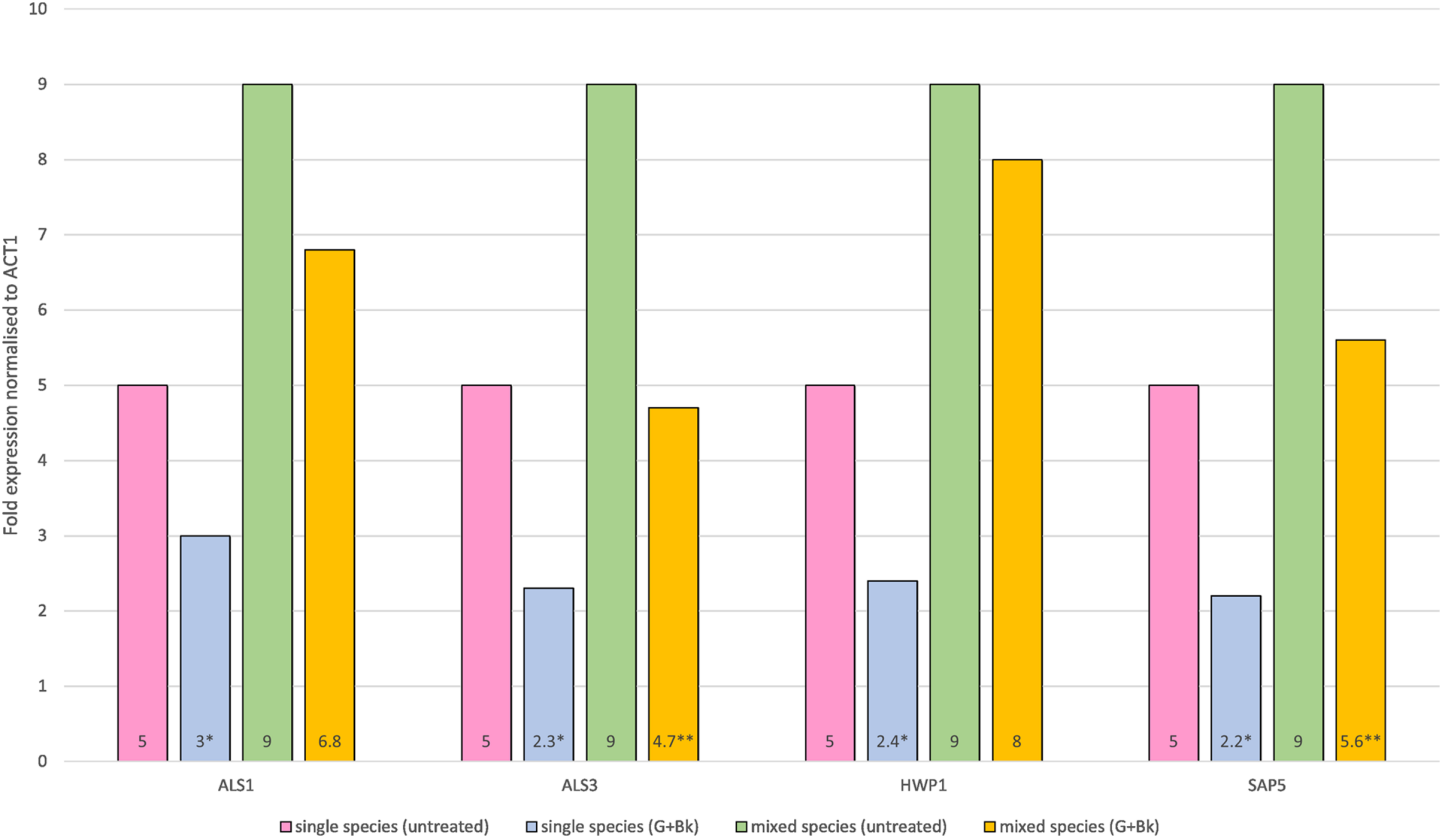
Relative gene expression of *ALS1, ALS3, HWP1* and *SAP5* in single species (*C.a* alone) and mixed species (*C.a+S.s+S.m*) biofilm after treatment with Bk+G. Means and standard errors based on triplicates are shown. An asterisk (*) denote value is significant in single species biofilm, *p* < 0.05; two asterisks (**) denotes value is significant in mixed species biofilm, *p* < 0.05.

In single species biofilm, the expression of all four genes was significantly decreased after treatment with Bk+G *i.e*., *ALS1* (*p* = 0.021), *ALS3* (*p* = 0.014), *HWP1* (*p* = 0.015) and *SAP5* (*p* = 0.01). In mixed species biofilm, the gene expression of *ALS3* and *SAP5* was significantly decreased (*ALS3*, *p* = 0.023 & *SAP5*, *p* = 0.033) (Fig. 1), whereas expression of *ALS1* and *HWP1* failed to decrease significantly (ALS1, *p* = 0.65 & HWP1, *p* = 0.74) (Fig. 1).

### Proteomic analysis

**Figure 2:**
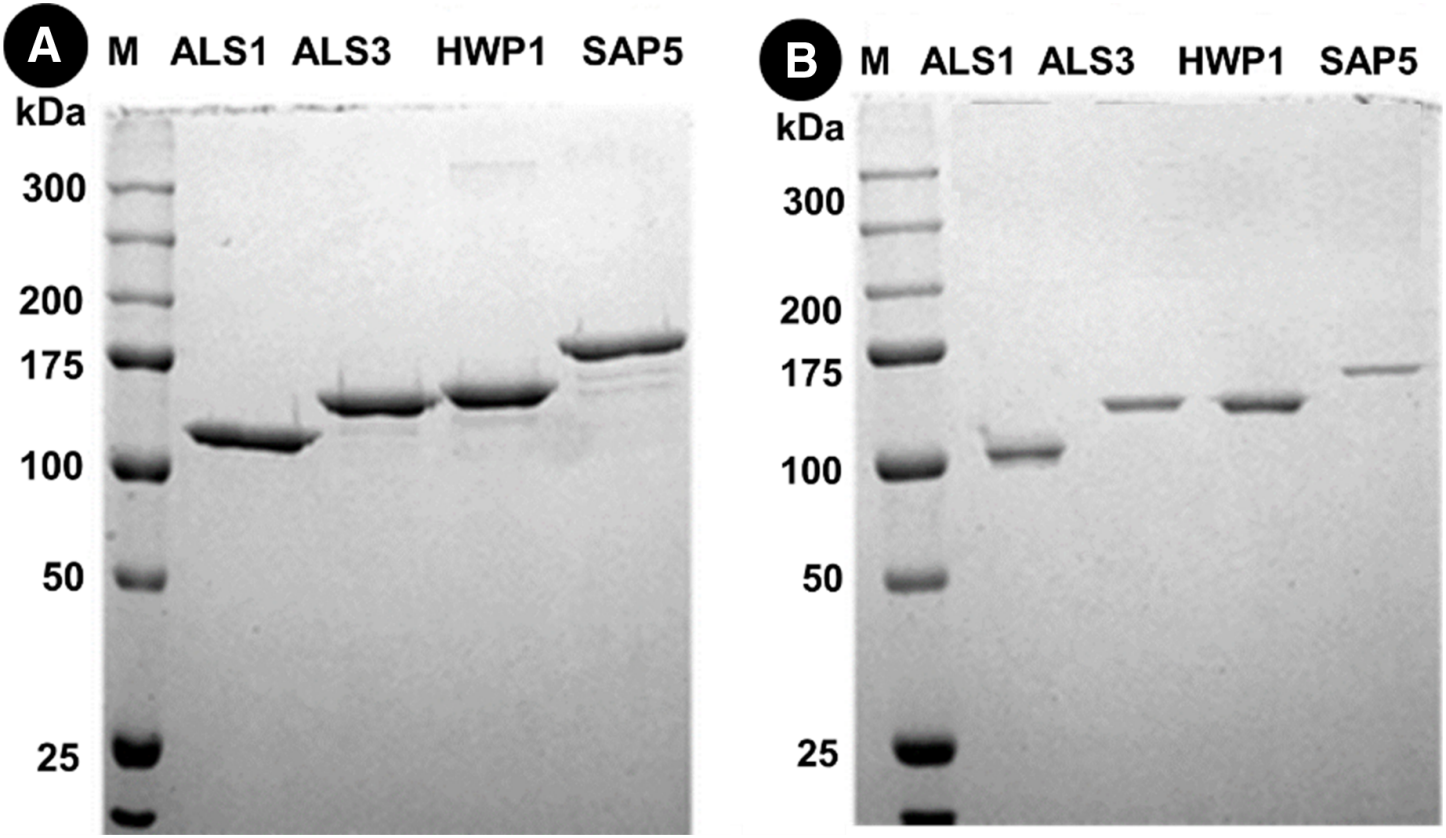
Western blot of candida cell wall proteins, before (A) and after (B) treatment with Bk+G in single species biofilm.

Protein separation followed by western blotting of proteins in single species (*C. albicans* alone) reveals thick bands of all four proteins *i.e*., als1p, als3p, hwp1p and sap5p with molecular weights ~100 kDA for als1p, increasing gradually for als3p (~145 kDA), hwp1p (~150 kDA) and finally to ~175 kDA for sap5p (Fig. 2A). It was observed that the respective bands were significantly decreased in width for samples treated with Bk+G (Fig. 2B).

**Figure 3:**
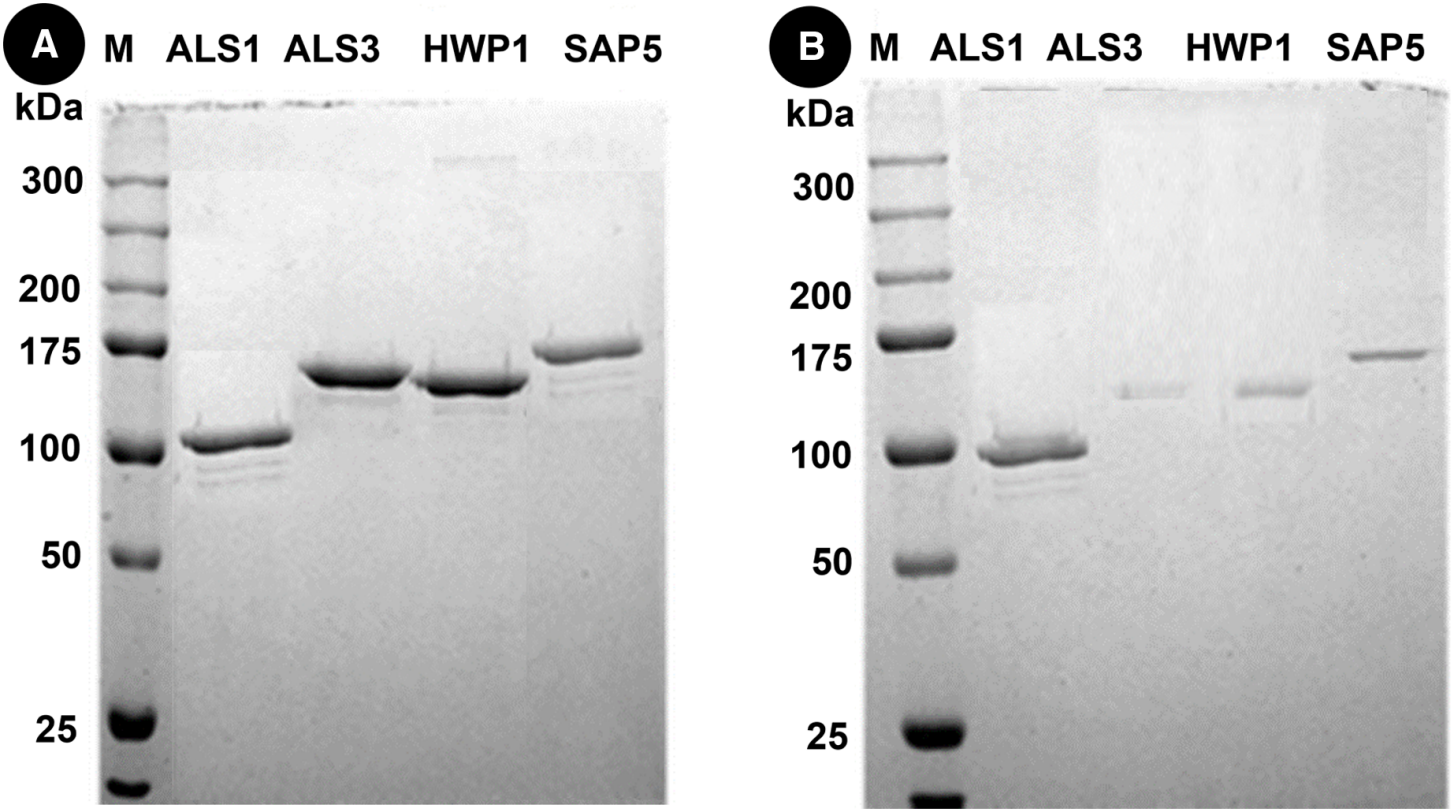
Western blot of candida cell wall proteins, before (A) and after (B) treatment with Bk+G in mixed species biofilm.

Western blots of proteins in mixed species (C.a+S.m+S.s) reveals thick bands of all four proteins *i.e*., als1p, als3p, hwp1p and sap5p with molecular weights of ~100 kDA for als1p, ~145 kDA for als3p, ~150 kDA for hwp1p and ~175 kDA for sap5p respectively (Fig. 3A). After treatment with Bk+G, the band formed with als3p and sap5p diminishes in width whereas band formed with als1p and hwp1p are still visible (Fig. 3B).

## Discussion

*Candida albicans* forms polymicrobial biofilms with a variety of bacteria, causing infections like denture stomatitis, periodontitis, oral candidiasis etc. which have increasingly become resistant to conventional antifungal therapy (Ponde et al., 2021). The problem of drug resistance encourages exploration of new therapeutics to combat polymicrobial biofilms.

Candida cell wall is responsible for adhesion of fungus with other microbes and host mucosa. It plays an important role in the pathogenicity of microorganisms and represents a possible target for inhibitors functioning as antifungal agents (Gow et al., 2017). Adherence, one of the key attributes of fungal cell-wall, is powered by both the presence of a salivary pellicle and specific adherence genes like *ALS, HWP* and *SAP* (Ardehali et al., 2019). Our previous study indicated that garlic extract, when used in combination with bakuchiol, inhibited the formation of candida polymicrobial biofilm *in vitro*. This study was conducted to investigate the effects of Bk+G extract on *C. albicans* by analyzing the gene expression, in an attempt to explore the action of reagents against virulence factors of *C. albicans*.

The crude extracts and metabolites derived from plants and herbs are valuable in the discovery of new antifungal agents (Samadi et al., 2019). *Bakuchiol* has been widely used to enhance skincare, as an anti-aging and anti-wrinkle agent and has previously displayed antimicrobial properties (Razak et al., 2018). Garlic extract can inhibit the growth of both Gram-negative and Gram-positive bacteria. The clove of garlic contains chemicals like alliin, allicin and ajoene (Mansingh et al., 2018; Reiter et al., 2020). When the garlic cloves are cut or crushed, they release the enzyme alliinase which converts alliin to allicin which is responsible for antibacterial activity (Nakamoto et al., 2020). The antimicrobial property of bakuchiol in combination with garlic extract was exhibited in previous studies which prove that these elements inhibit the growth of *C. albicans* alone and in combination with *S. sanguinis* and *S. mitis* (Fahim, Himratul-Aznita & Abdul-Rahman, 2019; Fahim, Himratul-Aznita & Abdul-Rahman, 2020).

The current study ought to expand on the previous findings that Bk+G is able to inhibit polymicrobial biofilm formation *in vitro*. The results indicate that Bk+G caused downregulation of all four genes in single species biofilm, whereas in polymicrobial species, Bk+G significantly reduced the expression of *ALS3* and *SAP5. ALS3* and *SAP5* are specifically involved in bud to hyphae transition of candida. *ALS3* is responsible for biofilm formation, adhesion to host tissue, iron acquisition and invasion of host cells (Liu & Filler, 2011). It is actively upregulated in candida mixed species biofilm. *SAP5* is involved in proteolytic activity of *C. albicans*. These proteinases are not only involved in invasive pathogenesis of candida, but they also facilitate candida adhesion to mucosal surfaces by degrading host barriers (Juvêncio da Silva et al., 2021). Downregulation of *ALS3* and *SAP5* indicates the role of Bk+G in reducing the virulence and subsequent dissemination capability of *C. albicans*. Downregulation of these genes may also inhibit covalent bond interactions of *C. albicans* with streptococci preventing polymicrobial biofilm formation as indicated by previous studies (Liu & Filler, 2011; Zhao et al., 2006). Researchers are now developing vaccines and antibodies against als3p to prevent candida infections (Sui, Yan & Jiang, 2017). Our study indicates downregulation of ALS3 with Bk+G and thus may prove useful in the synthesis of antifungal agents. However, further studies are required to determine why these genes are downregulated under the influence of Bk+G.

*ALS1* and *HWP1*, are although not directly involved in hyphae formation, they play a pivotal role in strengthening candida biofilm on oral surface by causing fungal adhesion to laminin and fibronectin of buccal epithelium, endothelial cells and monolayer cells (Goulart et al., 2018). Inhibition of all four genes in single species under treatment with Bk+G might suggest the loss of *C. albicans* biofilm firmness and stability on oral tissues. Previous studies have concluded that mutations in these genes causes lack of fungal biofilm formation (Mahmoudi, Amini & Amini, 2019). Bk+G was not able to downregulate *ALS1* and *HWP1* in mixed species biofilm. From this study, we cannot be certain as to why these genes resisted the effect. A possible explanation could be that Bk+G specifically targeted candida hyphae since it was able to downregulate *ALS3* and *SAP5* genes which are involved in hyphae formation (James et al., 2016). Another hypothesis is that polymicrobial biofilms have stronger bond strength between organisms in comparison to single species biofilm (Pathak, Sharma & Shrivastva, 2012; Ponde et al., 2021) and thus the multi species interaction may have prevented Bk+G to effect *ALS1* and *HWP1*. However, further studies are needed to conclude these hypotheses.

*In vivo* studies are required to ascertain the *in-vitro* antimicrobial effects of Bk+G combination and to adjust dosage for patients. These herbal agents are economical and locally available, and thus could effectively be incorporated in the formation of oral mouthwashes, gels or/and lozenges for the treatment of oral candidal and adjunctive infections.

## Conclusions

Our previous study indicated that biofilm biomass and cellular metabolic activity of *C. albicans* polymicrobial biofilm decreases under the treatment of *bakuchiol* and garlic. In this study, garlic extract and *bakuchiol* combination exhibited action against *Candida albicans* biofilms by significantly decreasing expression of important genes. The decrease in the expression of some genes could be related to the affectation in the virulence of Candida. But more experiments are needed to directly conclude that it has antifungal properties against *C. albicans*. The results propose possible future indication of these natural products for treatment of oral fungal diseases.

## Supplemental Information

10.7717/peerj.12251/supp-1 Supplemental Information 1 Raw data of Figure 1.

## Additional Information and Declarations

### Data Availability

The following information was supplied regarding data availability: The raw data of qPCR is available in the Supplemental File.
